# What lessons can African nations learn from the COVID-19 pandemic?

**DOI:** 10.1186/s41182-022-00480-x

**Published:** 2022-11-28

**Authors:** Olanrewaju Akinola, David Bamidele Olawade, Aanuoluwapo Clement David-Olawade

**Affiliations:** 1 School of Law, Politics and Sociology, The University of Sussex, Brighton, UK; 2 Center for Population and Reproductive Health, University of Ibadan, Ibadan, Oyo Nigeria; 3 Epsom and St Helier University Hospitals, NHS Trust, Carshalton, UK

**Keywords:** COVID-19, Economy, Health, Africa

## Abstract

While the impact of the COVID-19 pandemic differed per country, the impact on African nations was comparable. Health, money, the economy, education, and inventions have all been criticised over the years, but the discoveries during the pandemic were stunning. We witnessed a system being devoured by corruption and anti-patriotic citizens seeking their agenda. The commentary discusses the influence of the COVID-19 pandemic on several sectors in African nations, as well as the lessons that may be drawn from it. Furthermore, it recommends methods to avoid a recurrence.

## Introduction

The World Health Organization (WHO) Director-General designated the coronavirus illness (COVID-19) epidemic a worldwide public health emergency of international concern on January 30, 2020, by the International Health Regulations (2005) [1]. Africa experienced its first case on 14 February, in Egypt, and COVID-19 was declared a global pandemic on 12 March [2], which later spread across many other African nations within months. The COVID-19 pandemic taught Africans a lot about the system they are building and how it is not sustainable. The state governments worked hard to curtail this virus, ‘nonetheless be under no illusion the catastrophic impact that poorly managed COVID-19 control measures are having on education, food security, access to safe water, health and jobs, with a real absence of measures to mitigate the impact on some of the poorest people on the Continent’ [3]. In May 2020, the United Nations Economic Commission for Africa (UNECA) estimated that a one-month full lockdown across Africa would cost the continent about 2.5 per cent of its annual gross domestic product (GDP), i.e. approximately US$65.7 billion per month [4].

## Health

The principal challenge for African countries is their fragile and strained public healthcare systems. African countries are overly dependent on other nations for pharmaceutical products [5]. The COVID-19 pandemic exposed the inadequacy, corruption-filled and dilapidated state of the health sectors in African countries. In comparison with some other infectious diseases, coronavirus does not rate so highly with impact on mortality rate although it had a wide range across the world in 2020. Ebola has a mortality rate of about 50%, making it a very lethal disease. However, there were few ways for it to spread because only direct contact with human fluids like blood or perspiration could do it. Health experts claim that 2019-CoV has developed the capacity to easily spread between individuals and can do so even before symptoms manifest. It has, however, given up part of its strength since, at 15%, it has a significantly lower death rate than Ebola [6]. However, it has made plain the weak state of things in African countries.

The healthcare sector should learn that physicians and nurses need better tools to perform their duties. The continent of Africa has to strive to provide a secure workplace for those who work in this industry. Only a few fully functional government hospitals with the necessary equipment could be found in Nigeria, which was sad enough. In addition, private hospitals refused to accept patients because they had no idea what to do and could only offer preventative measures to avoid coming into contact with the virus.

In African countries, there is a need to focus on adequately teaching practitioners, and by training, I mean that provisions should be made accessible for health workers to feel comfortable and operate in a working environment. The overdependent nature of African countries on international communities for help without having taken the basic steps is alarming. For example, it was discovered that 10 African countries have no ventilators and even the ones that do have had limited ventilators which could not cater for the needs of the people [7]. To buttress my point, it was noted that ‘getting more ventilators to African countries is not enough, though. Trained medical personnel are also needed to run the machines, as well as a reliable electricity supply and piped oxygen’ [8]. A further factor affecting the health sector is research. Research facilities in African countries are inadequate, and research is not conducted methodically or has never received global recognition, which is why when a vaccine was developed, it did not receive much national recognition, let alone international recognition because the processes are not trusted. Dr Christian Happi, a molecular biologist and genomicist who directs the African Center of Excellence for Genomics of Infectious Diseases (ACEGID) in Nigeria, was questioned if vaccines are being developed in Africa. "The fact is, yes they did," he said. But were those vaccine candidates backed up? "Africa did not invest in COVID-19 vaccine development when we could have produced a vaccine for the African population," says the response [9].

## Economic impact

COVID-19 revealed the poverty state of African countries; we saw an increase in poverty levels during the pandemic, as people were found helpless and without adequate means of provision to survive. This is due to improper planning, a high rate of unemployment and inadequate financial planning for many families [10]. The oil-producing countries like Nigeria, Angola, Algeria Libya, Egypt, Sudan, the Republic of Congo, Equatorial Guinea, and Ghana saw a big fallout in the first quarter of 2020, oil prices fell by 50 per cent, the South-South African countries that deal with the export of raw minerals also saw there destabilising effects of COVID-19 has prices fell [8, 11]

The countries that prioritise tourism, like South Africa, Rwanda, Botswana, Seychelles, Tanzania, and Kenya, also received a fair shake in this situation because employment and state revenue were both impacted. As consumable goods are imported, leaving everyone at the mercy of importation during the pandemic, most borders were closed, which made the situation even worse, and citizens had to revert to locally produced food that had not undergone the proper processing, there is a need to start encouraging massive production in the African countries. It has been suggested that the government begin to take use of the robust agricultural industry in each region since this would aid in reducing poverty.

## Innovation

When the whole Financial Technology (FinTech) Movement began in African countries in 2013, it did not appear to be the in-thing; rather, it was viewed as the financial industry's future. However, the COVID-19 epidemic brought home to everyone just how important it was. The people had to rely on these methods of exchange for money. However, Africa as a whole was exposed to its bad network connections, which had a significant impact on many areas and sectors, including the judiciary, where cases were postponed owing to the poor connection, and you find attorneys, judges, and clients being agitated by the same.

Additionally, schools were closing around this time (particularly the government-run institutions), which meant that students had to automatically extend their study periods without being adequately compensated or receiving a formal apology. According to a UNICEF study from November 2020, 250 million children in sub-Saharan Africa have been prevented from attending school due to COVID-19-related school closures [12]. The school that managed to hold exams online had various complaints from students ranging from poor networks, unfriendly user interface, lack of proper planning, etc. [13].

It is necessary to promote more technologically advanced and digitalised settings in the fields of health, education, and business to hasten the development of these nations. Today, using technology is the new norm for a variety of tasks; even private businesses are transferring all of their work from paper to digital environments for a more productive and efficient labour.

## Conclusion

Although the COVID-19 epidemic began in 2020, we are still struggling with its after-effects in 2022 and we are using all of our resources to combat it. Even though some states have been able to recover from some of its effects, it is advised that African countries start investing in the healthcare industry and creating a technologically advanced environment, with a focus on making the best investments possible with borrowed money so that investment returns can be used to pay off debts. As Dr Akinwumi Adesina has stated, "Social distance is a necessity, but budgetary distancing is not", there is also a need to focus on renewable energy (such as the solar system, and recycling of garbage) as this would save money and strengthen the economy. This requires investment [14].

## Data Availability

Not applicable.
